# Functional neurological restoration of amputated peripheral nerve using biohybrid regenerative bioelectronics

**DOI:** 10.1126/sciadv.add8162

**Published:** 2023-03-22

**Authors:** Amy E. Rochford, Alejandro Carnicer-Lombarte, Malak Kawan, Amy Jin, Sam Hilton, Vincenzo F. Curto, Alexandra L. Rutz, Thomas Moreau, Mark R. N. Kotter, George G. Malliaras, Damiano G. Barone

**Affiliations:** ^1^ Electrical Engineering Division, Department of Engineering, University of Cambridge, Cambridge, UK.; ^2^ Department of Clinical Neurosciences, University of Cambridge, Cambridge, UK.; ^3^ Bit Bio, Cambridge, UK.

## Abstract

The development of neural interfaces with superior biocompatibility and improved tissue integration is vital for treating and restoring neurological functions in the nervous system. A critical factor is to increase the resolution for mapping neuronal inputs onto implants. For this purpose, we have developed a new category of neural interface comprising induced pluripotent stem cell (iPSC)–derived myocytes as biological targets for peripheral nerve inputs that are grafted onto a flexible electrode arrays. We show long-term survival and functional integration of a biohybrid device carrying human iPSC-derived cells with the forearm nerve bundle of freely moving rats, following 4 weeks of implantation. By improving the tissue-electronics interface with an intermediate cell layer, we have demonstrated enhanced resolution and electrical recording in vivo as a first step toward restorative therapies using regenerative bioelectronics.

## Teaser

Implanted human iPSC-derived myocytes with flexible electronics on peripheral nerves allow enhanced electrophysiology recording.

## INTRODUCTION

A major hurdle in reversing the effect of injury to the peripheral nervous system is the inherent inability of neurons to regenerate and to rebuild disrupted neural circuits. Implantable neurotechnology and cell therapy are rapidly developing as potential effective treatments. These methods attempt to restore function by either bypassing the injury site and electrically interacting with existing neurons or providing new cells to replace the damaged ones. Unfortunately, they both have drawbacks, which have slowed down their translation to the clinic. In the context of damaged tissue in the mature nervous system, transplanted neurons struggle to reestablish functional connections in existing circuits without appropriate guidance (*1*). Similarly, electrodes cannot work without healthy working cells to interface, either because these cells are compromised by the injury or hidden by the formation of dense scar tissue around the implant [i.e., foreign body reaction (FBR)] (*2*–*7*). Moreover, current neurotechnologies lack the selectivity and specificity to interface to different subtypes of neurons responsible for different functions (*8*). Personalized electronic therapies (*3*) are being developed to include biologically inspired materials to treat nervous system injuries.

A critical limiting factor is the resolution with which nerve inputs are mapped onto implants (*3*). This is determined by a variety of factors such as proximity between electrically active cells and electrodes, as well as the amplitude of their signals (*9*). A biohybrid strategy incorporating cells as an intermediate layer on electronics allows for a “controllable” synaptic integration between implanted cells and existing neural circuitry. Biohybrid implants have the potential ability to host, interact, and control the behavior of transplanted cells; promote organized, functional cellular integration with living tissue; and reduce scar tissue formation (i.e., FBR) (*10*, *11*). We hypothesized that the use of a scalable cell source, which can be integrated into a bioelectronic device as a biological target for peripheral nerve inputs, may allow for recording from selected subsets of nerve fibers, decrease axon-electrode distance, and improve signal amplitude, potentially increasing spatial and neuron class recording resolution. On the basis of these principles, we report the development of a novel biohybrid neural interface, combining long-term surviving induced pluripotent stem cell (iPSC)–derived human skeletal myocytes and flexible electronics in a chronic sensorimotor nerve rat model and demonstrating tissue integration and enhanced electrophysiology recordings.

## RESULTS

**Fig. 1.**
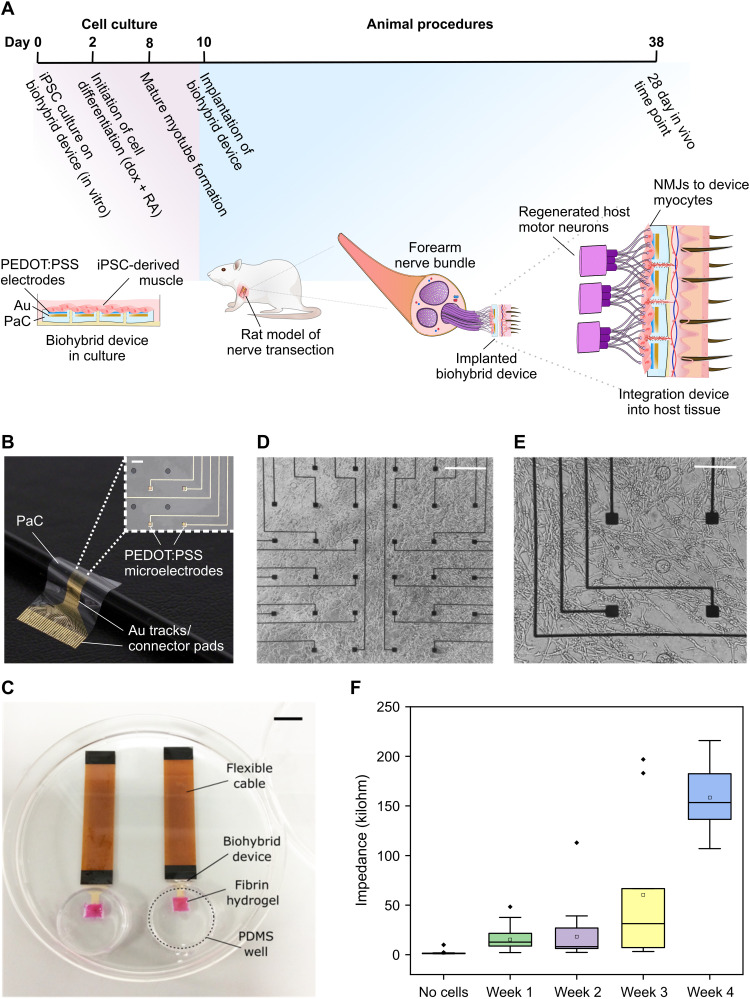
A biohybrid peripheral neural interface. (**A**) Experimental timeline showing the fabrication and implantation of the biohybrid device from an in vitro cell culture step into an animal model. Cells are seeded onto the flexible biohybrid devices at day 0. After 48 hours (day 2), the differentiation process is initiated. At day 8, myotubes are mature; therefore, between day 8 and 10 is the optimal timing for the implantation of the biohybrid devices into a peripheral nerve rat model. Devices are then implanted for a period of 4 weeks. (**B**) An in vivo biohybrid device design customized for the peripheral nervous system. Devices consist of two PaC, parylene-C, layers containing Au tracks and PEDOT:PSS microelectrodes. Au tracks lead to connector pads to which a connecting flexible cable can be bonded. The parylene-C layers contain holes to permit fluid flow and cell migration across the device. Scale bar, 60 μm. (**C**) A completed biohybrid device, including a fibrin hydrogel and iPSC-derived muscle cells. Scale bar, 1 cm. (**D** and **E**) A bright-field image showing human iPSC-derived myocytes at day 8 of culture. Scale bars, 465 μm (D) and 230 μm (E). (**F**) Electrode impedance at 1000 Hz before and after cell addition over a 4-week period postimplantation. PaC, parylene C; Au, gold; dox, doxycycline; RA, retinoic acid; NMJs, neuromuscular junctions; PDMS, polydimethylsiloxane.

The first step in the development of the biohybrid device was to choose an appropriate timeline to culture and sufficiently mature cells in vitro before implanting the device in vivo. We chose skeletal myocytes generated from human iPSCs by OPTi-OX cellular reprogramming as the biohybrid cell population as this system consistently produces highly pure myocytes after 8 days of culture (*12*). This made them well-suited to host sensorimotor nerves, whose motor axons typically innervate muscle tissue, while human iPSC-derived cells have the potential to provide off-the-shelf cell material for future clinical applications (clinically translatable). On the basis of the properties of the iPSC-derived myocytes and of injured nerves, which typically regenerate within 3 weeks after injury (*13*), we determined a timeline to test the ability of biohybrid devices to integrate with host nerves (Fig. 1A).

We cultured the iPSCs on thin, flexible parylene-based microelectrode arrays (MEAs). The MEAs were fabricated using standard photolithography techniques (*14*) to contain 32 conducting polymer [poly(3,4-ethylenedioxythiophene) polystyrene sulfonate (PEDOT:PSS)] electrodes arranged in a symmetrical grid. The MEA occupied a 2-mm by 2-mm area within the larger parylene device, within which we introduced circular openings to permit growth of vasculature from the back of the device and support cell survival postimplantation (Fig. 1B).

The culture process consisted of iPSC clusters being seeded on a fibrin hydrogel layer deposited on the MEA surface to help encapsulate the cells on the device (Fig. 1C). Following the induction of reprogramming 48 hours later, mature multinucleated myotubes are formed on the surface of the biohybrid device by day 8 (Fig. 1, D and E; culture process summarized in fig. S1), which, along with the acetylcholine, induced contractions (fig. S2), demonstrated myocyte maturity (*12*, *15*). The biohybrid devices show good stability over the timeline used in the study, exhibiting an acceptable and expected increase in impedance over 4 weeks of in vivo culture (before cell seeding: 97% yield, 1.84 ± 2.20 kilohm; week 4 in vivo: 25% yield, 159.00 ± 35.80 kilohm, means ± SD) (Fig. 1F).

The biohybrid devices containing mature myotubes were then implanted into immunodeficient rats to enhance human myotube survival, representative of systemic immunosuppression in humans, a strategy commonly used in cell transplantation studies (*16*–*18*). Biohybrid devices were implanted under the skin of the animal, with the MEA and cells facing the underlying musculature. The dermal layer onto which the device was laid was scored with a sterile knife immediately before device implantation to promote tissue regrowth and angiogenesis in the vicinity of the implant. We confirmed that this implantation strategy could support cell survival for 7 days after implantation (fig. S3).

**Fig. 2.**
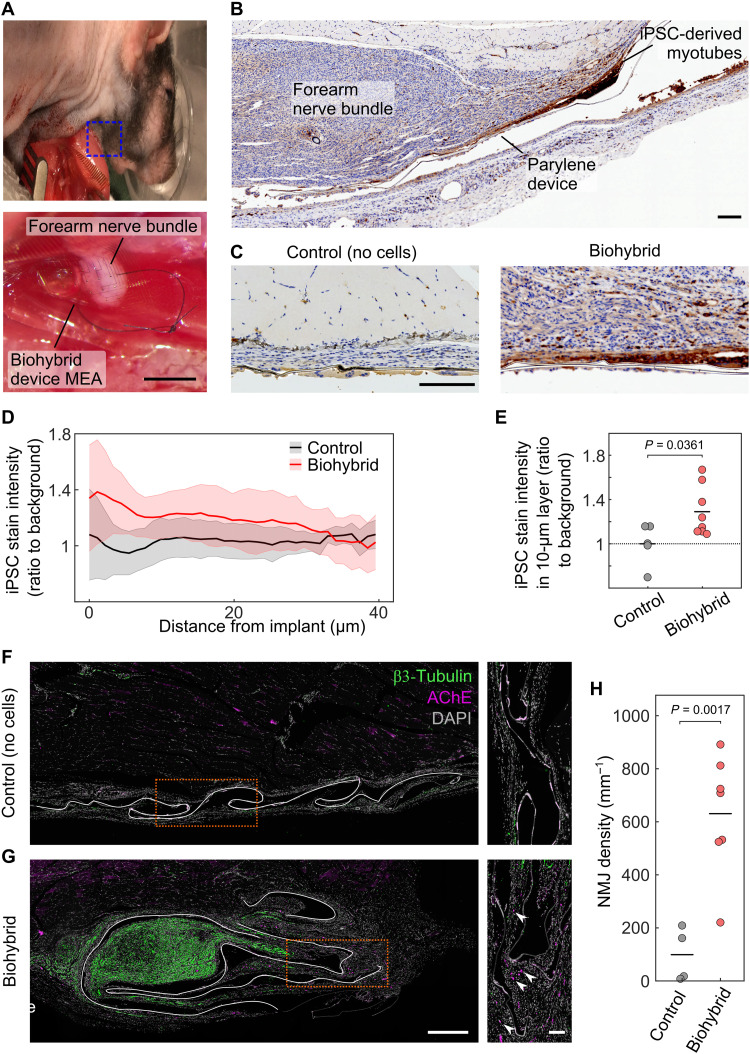
Integration of injured host nerve with iPSC-derived muscle cells on a biohybrid device in a 28-day chronic rat study. (**A**) Surgical implantation of our biohybrid device into the rat forearm nerve bundle. Scale bar, 2 mm. (**B**) Image of human nucleoli stained (red/brown) biohybrid device after 28 days of implantation. Survival of a layer of human iPSC-derived muscle cells visible as a layer of stain near the parylene of the device. Scale bar, 50 μm. (**C**) Magnified images of control (lacking iPSC cells) and biohybrid implants 28 days postimplantation. The high cell density tissue in proximity to biohybrid image is part of the regenerated forearm nerve bundle. The tissue near the control image corresponds to musculature under the device. (**D**) Human nucleoli stain intensity (ratio to background) over distance from the implant in both the control (black) and biohybrid device implants (red) (means ± SD). (**E**) Average human nucleoli stain intensity in the 10-μm layer closest to the implant. Biohybrid devices showed a significantly enriched presence of iPSCs compared to control devices. (**F** and **G**) Images of immunofluorescence stains of biohybrid and control devices (lacking cells) for axons (β3-tubulin, green) and NMJs [AChE (acetylcholinesterase), magenta], as well as cell nuclei [DAPI (4′,6-diamidino-2-phenylindole), gray] 28 days postimplantation. The host nerve is found in close proximity to biohybrid but not control devices. An abundance of NMJs are seen in immediate proximity to biohybrid but not control devices (white arrowheads in inset indicate examples of NMJs). The position of the implant is marked by a white line in images on the left. Scale bars, 500 μm (left) and 100 μm (insets). (**H**) Quantification of NMJ density. (E and H) Circles in the plot represent average in individual rats; black bars indicate the mean of the group. *P* values were calculated via Student’s *t* test.

We then examined the ability of our biohybrid devices to host and integrate with a regenerating nerve. We chose the ulnar and median nerves, which control sensory and motor paw function and run together through the arm of the animal, hereafter referred together as forearm nerve bundle. This choice was driven by the clinical relevance of injury to upper limb nerves and higher degree of fine motor and sensory functions. Implantation was carried out by transecting the nerve bundle at elbow height and suturing the proximal nerve stump to the cell-laden side of the biohybrid device. The device was then transferred a few centimeters toward the midline of the animal and anchored subcutaneously above the latissimus dorsi muscle (Fig. 2, A and B). iPSC-derived cells were found to survive following 4 weeks of implantation with the sutured nerve (Fig. 2, C to E). The transplanted cells remained closely adhered to the biohybrid device following this period (Fig. 2, D and E). Immunofluorescence stains of the tissue surrounding biohybrid devices indicated the presence of neuromuscular junctions (NMJs) on the surface of the biohybrid devices, but not of control devices lacking myocytes (Fig. 2, F to H), and suggested the innervation of the device by host motor axons. Although no major differences in host tissue appearance and cell density were seen across biohybrid and control devices (Fig. 2, B and C and fig. S4), the nerve to which the device was sutured was often found in very close proximity to biohybrid devices (Fig. 2, B, C, and G), but not control devices (Fig. 2, C and F).

**Fig. 3.**
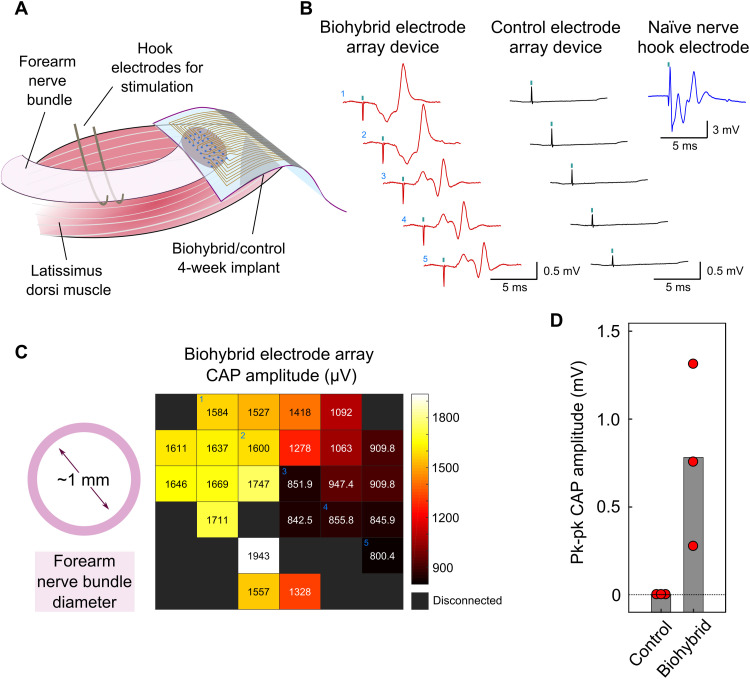
Biohybrid devices enable transected nerves to retain healthy nerve electrophysiology 28 days postimplantation. (**A**) Schematic of the experimental setup. Twenty-eight days postimplantation, forearm nerve bundles transected and implanted with either biohybrid or control (lacking iPSCs) devices are exposed. Under anesthesia, the nerve response to electrical stimulation is evaluated using the chronically implanted devices. (**B**) Representative traces of nerve response to 100-μA, 0.1-ms stimulation pulses, taken from different electrodes within a device MEA. CAPs are recorded from biohybrid (red) but not control (black, lacking a culture of iPSC-derived myocytes) devices. CAP was recorded from a naïve (nontransected) nerve using hook electrodes shown in blue for comparison. Stimulation time is identified by cyan squares in traces. CAPs recorded by electrodes within the biohybrid MEA each likely from a segment of the nerve cross section are similar to those recorded from a whole intact nerve.(**C**) Average pk-pk CAP amplitude from the MEA of a the biohybrid device from (B). Higher CAP voltages represented by lighter colors in the heatmap, with exact values represented by the numbers. Electrodes with impedance of >500 kilohm are considered disconnected and shown in black. The approximate dimensions of the forearm nerve bundle are indicated by the magenta ring. Nonuniformness of CAP recordings with higher amplitudes loosely matching dimensions of the nerve is suggestive of selective recordings of the nerve from the device MEA. Individual electrodes from which the traces in (B) are taken are indicated by cyan numbers 1 to 5. (**D**) Quantification of pk-pk CAP amplitude in 28-day implanted biohybrid and control devices for 100-μA pulse stimulation. Control devices exhibit no CAPs in response to stimulation, while all biohybrid devices do. Red circles represent values for each animal (mean across entire MEA); the bar indicates the mean of the whole group.

Good integration of the host forearm nerve bundle with the transplanted iPSC-derived myotube population was expected to result in improved nerve signal quality as recorded by the biohybrid implant. We recorded nerve signals from nerves implanted with biohybrid devices and control devices lacking cells 4 weeks after implantation, the time point by which nerve regeneration and integration into the nearby biohybrid cell population was expected to have occurred. At this time point, we exposed the connections of the implanted biohybrid/control device MEAs and placed a pair of hook electrodes around the forearm nerve bundle approximately 4 cm above the point of transection. Using the hook electrodes, we electrically stimulated the nerve and recorded the response using the 4-week implanted devices (Fig. 3A). We stimulated the nerve using a 0.1-ms duration pulse, for which a threshold of activation of 100 μA had been previously measured in the contralateral forearm nerve bundle. When the implanted nerve was electrically stimulated with a 100-μA pulse, a compound action potential (CAP) was recorded from biohybrid but not control implants (Fig. 3B). This CAP consisted of a peak with an approximately 2 ms delay (corresponding to a conduction speed of ~20 m/s), consistent with Aα/β fiber activation, followed by a later peak, likely corresponding to reflex activity initiated by sensory fiber activation (H-reflex). These CAP features are consistent with those found in intact sensorimotor nerves (Fig. 3B) (*19*, *20*), indicating healthy nerve physiology in the transected forearm nerves implanted with a biohybrid device. The amplitude of the CAPs recorded by the biohybrid MEA electrodes was lower than seen in hook electrodes around intact nerves, likely as a consequence of the smaller size of the former and indicative that these are each recording from only a portion of the nerve.

CAPs recorded over the entire biohybrid MEA significantly differed in amplitude. MEAs were designed with dimensions of 2 mm by 2 mm, larger than the forearm nerve bundle (diameter of ~1 mm). This was designed to allow for the identification of different features in the recordings of electrodes under the nerve compared to those around it (Fig. 2A). Hotspots in CAP amplitude in the MEA corresponded in dimensions to the forearm nerve diameter (Fig. 3C and fig. S5), further supporting that the nerve had integrated with the biohybrid device over the implantation period. While all nerves implanted with biohybrid devices produced CAPs in response to 100-μA stimulation pulses, their average amplitudes differed, suggesting a degree of variability in biohybrid device integration across animals (Fig. 3D). Consistent with normal nerve behavior, stimulation with pulses of amplitude below activation threshold resulted in no CAP (fig. S6). Stimulation with much higher amplitude pulses eventually resulted in CAPs recorded in both groups (fig. S6). However, CAPs recorded by control devices under these stimulation conditions consisted exclusively of an H-reflex, which may have been mediated by reflex activity of other nerves at the same cervical level as the forearm nerve bundle.

**Fig. 4.**
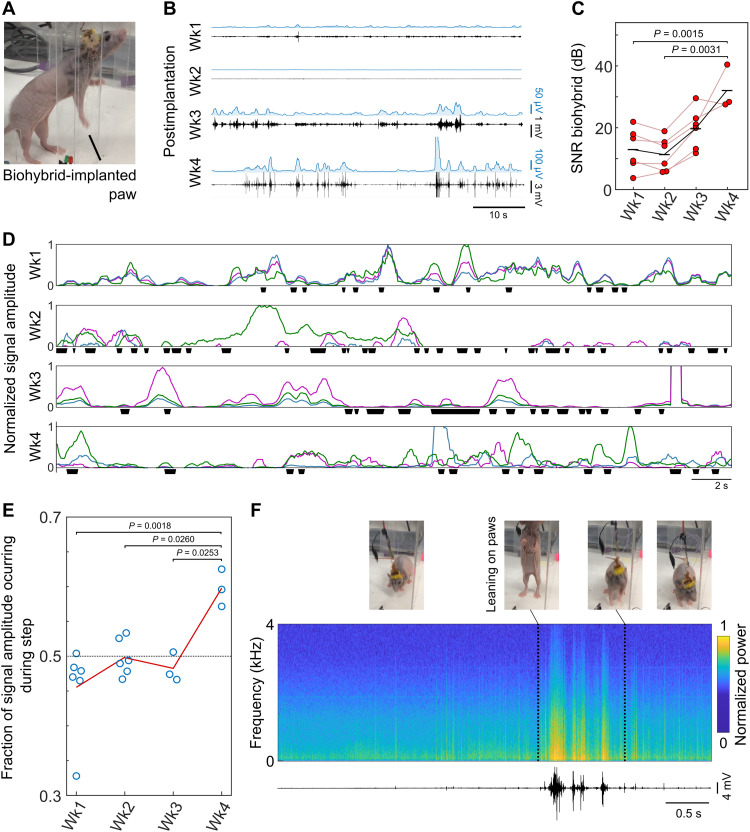
Nerve electrical recordings from biohybrid devices progressively improve over 4 weeks postimplantation, coinciding with nerve regeneration. (**A**) Photograph of experimental setup. Rats are implanted with a biohybrid device into their right forearm nerve bundle, with the connections externalized through a headcap. This permits awake recordings over weeks postimplantation. (**B**) Forearm nerve signal traces recorded from biohybrid devices over 4 weeks postimplantation. Black, band-pass–filtered (0.2 to 4 kHz) time traces recorded from a pair of electrodes in the MEA (bipolar configuration); cyan, root mean square (RMS) of black traces with 0.5-s rolling window. Nerve signal amplitude increases over the implantation period. (**C**) Quantification of SNR of recorded traces. Red circles, mean value per bipolar electrode; black line, mean of group; *n* = 2 rats. Recordings were taken from the same electrodes throughout experimental timeline. Statistical comparison was done via one-way analysis of variance (ANOVA) followed by Tukey post hoc test. *P* values not shown are >0.05. (**D**) RMS time traces from three bipolar electrodes (cyan, green, and magenta) over 4 weeks of implantation, normalized to range from 0 to 1 for each week. Stepping with the implanted paw indicated by black squares. Increased correlation between recorded activity and paw movement, indicative of good forearm nerve recording, develops at week 4 postimplantation. (**E**) Quantification of correlation with stepping. Circles, mean normalized RMS occurring during steps; line, mean of the group; *n* = 2 rats. Statistical comparison was done via one-way ANOVA followed by Tukey post hoc test. *P* values not shown are >0.05. (**F**) Sample time-frequency spectrogram and trace of a recording 4 weeks postimplantation. High activity is seen when a rat leans on a chamber wall (paw leaning delimited by black dotted lines), compared with lower activity when the animal remains on the bottom of the chamber, indicating that activity is driven by attempted paw use.

To track the integration of nerve with the biohybrid device, we implanted two animals with biohybrid devices in a similar fashion and externalized the MEA connections through a headcap. Over the course of 4 weeks, we performed weekly recordings through the implanted biohybrid device while the animal roamed freely in an arena (Fig. 4A and fig. S7). To minimize electromyogram noise from nearby musculature, we set up bipolar recording electrodes between pairs of electrodes across the biohybrid MEA. We consistently maintained these pairs throughout the 4 weeks of implantation. Within the first 2 weeks of implantation, we observed little activity in the awake animals. By the third and, in particular, the fourth week, signals had greatly and significantly increased in amplitude [weeks 1 to 4: 12.9-, 11.3-, 19.7-, and 32.0-dB mean signal-to-noise ratio (SNR), respectively] (Fig. 4, B and C). The improvement was consistent and uniform within each device, with every electrode examined showing an increase in SNR by week 3 (Fig. 4C). As axon regrowth usually begins 4 days after injury (*21*), and accounting for the small distance between nerve and biohybrid device, the recorded increase in signal amplitude matched the expected timeline of axon regrowth into the biohybrid device.

The integration of the forearm nerve bundle into the biohybrid device was expected to result in an activity that correlated with paw function, normally driven by this nerve. Therefore, next, we evaluated the correlation of electrical activity recorded by the device with paw function while the animal roamed around the cage. The forearm nerve bundle controls paw function. However, as it had been transected as part of the implantation procedure, paw or digit movement could not be used as an indicator of nerve activity. We instead identified stepping movements, which would normally end with the extension of the digits as the paw approaches a surface and matched these events to record electrical activity (Fig. 4D). We observed that, at early implantation time points, recorded activity appeared to be independent of movement of the operated limb. However, by week 4 of postimplantation, activity increasingly correlated with movement of the implanted paw (Fig. 4, D to F). We also observed that, by week 4, different electrode pairs often recorded activity independently of each other (Fig. 4D). This may indicate that different axon bundles of the forearm nerve bundle, which typically innervate different muscle groups around the paw, had grown into different regions of the MEA consistent with the detected NMJs in the biohybrid devices (Fig. 2F). This is further supported by the large increase in activity amplitude that developed at this time point (Fig. 4, B and C). Activity at earlier time points was instead typically recorded across all electrodes. Other changes, such as a moderate increase in electrode impedance, were also observed between week 4 and previous time points (Fig. 1F). These observations support that, following 4 weeks of implantation, the host nerve had grown into and integrated with the biohybrid device, greatly improving recording performance.

## DISCUSSION

We have introduced a new category of neural interface, which combines flexible electronics and human iPSC-derived cells. We have shown how implanted human iPSC-derived myocytes can survive in a rat model for up to 28 days and integrate with the host tissue forming NMJs and can be used to restore and drive functionality through a biohybrid device. The biohybrid neural interfaces showed superior electrophysiology recording properties and tissue integration compared to standard neural interfaces (flexible electronics without cells). This novel strategy enables axon fiber type–specific recording selectivity and potentially significant increases in spatial resolution and opens the door to the development of the next-generation biohybrid prosthetics as treatments for severe clinical conditions involving peripheral nerve damage (i.e., amputations).

Recent advances in neural interfacing technologies have produced devices for the restoration of function in amputees. Some recent systems tested in human patients make use of brain recording interfaces to acquire and interpret motor signals from the patient cortex and drive a robotic prosthesis (*22*, *23*). Alternatively, penetrating nerve implants can be used to stimulate sensory axons within the nerve stump to recreate sensory perception in the amputated limb (*24*, *25*). However, these approaches either require complex patient-specific interpretations of cortical activity to be associated with muscle movements or are limited to the restoration of sensory perception but not motor control. Interfaces capable of recording directly from nerves following amputation have also been developed (*26*, *27*). These regenerative interfaces typically permit axon growth through channels or holes within their architecture to produce a high-quality interface to record signals from axons. Most of these regenerative nerve implants, however, have seen little translation to human use in part due to substantial loss of performance at long (>6 months) time points (*26*, *28*). This has been associated with buildup of fibrotic tissue due to FBR, which clogs the channel lumen and damages axons (*28*).

The biohybrid devices we have developed offer unique advantages in the context of amputation treatments by providing higher signal quality through the biological amplification step performed by the innervated myocytes. Moreover, the selection of the transplanted cell type offers a unique mechanism to interact with a specific type of axon—in the case of the biohybrid system presented here, recording from motor axons specifically which could be implemented for the control of a motor-driven prosthesis. The independent communication with axons transmitting different types of information may enable more flexible control and sensation in prosthetic systems, greatly improving their application in amputations.

While biohybrid devices conceptually rely on regeneration to integrate with host tissue, our devices used a minimalist two-dimensional (2D) design fabricated from ultraflexible materials and containing a cell biohybrid layer, two factors associated with greatly decreased FBR (*2*, *10*). Moreover, as the regenerative design implemented did not require axons to regenerate through the implant body itself, we expect the biohybrid system presented here to avoid the long-term stability issue encountered by other regenerative designs.

The timeline of the appearance of high signal amplitude recordings in the developed biohybrid devices provides an informative outline of the tissue integration events occurring around these devices in vivo. The first week following nerve transection and device implantation yields low-quality signals as damaged axons retract and begin regenerating. The presence of a small, millimeter-size gap between the nerve stump and device created during implantation will lead to the growth of a nerve scaffold to serve as a bridge before axon regeneration crossing it takes place (weeks 1 and 2) (*29*, *30*). Arrival of axons to the proximity of electrodes may produce an increase in signal amplitude (week 3). However, NMJ formation with biohybrid myocytes will have to occur before myocytes summate their electromyogram activity to that of axons to improve signal amplitude, a process which may last over a week (week 3 to 4) (*31*, *32*). The biohybrid signal recording evolution (Fig. 4) and integration of nerve and implant at 4 weeks postimplantation (Fig. 2, F to H) support this timeline of events.

Looking ahead at the wider impact of this technology, biohybrid neural interfaces could be adapted through the use of different transplanted cell types such as those with neuronal or glial phenotypes to promote integration with other tissues such as brain and spinal cord. This could potentially extend the scope of treatments addressable by this technology to conditions such as stroke, traumatic brain injury, and spinal cord injury. By selecting the appropriate implant design and cell type, customizable biohybrid neural interfaces could be generated to meet patients’ individual requirements. Furthermore, the combination of an implanted device and cells allows, through bespoke genetic modifications of parental iPSC, for instance, for the use of local drug delivery for immunosuppression or growth factor delivery.

## MATERIALS AND METHODS

### Device fabrication

A 2-μm-thick parylene C layer was deposited on silicon wafers (100 mm of outer diameter, thickness of 1 mm) using an SCS Labcoater 2. Gold electrodes and interconnects were patterned through a metal liftoff process. An AZ9260 photoresist was spin-coated at 3500 rpm on the substrate, baked at 110°C for 120 s, exposed to ultraviolet light using a Karl Suss Contact Mask Aligner MA/BA6, and developed with an AZ 760MIF developer. A 10-nm-thick Ti adhesion layer, followed by a 100-nm-thick Au layer, was deposited (Angstrom EvoVac Multi-Process) and patterned by soaking the substrate in a bath of acetone for 10 min. A second 2-μm-thick layer of parylene C (insulation layer) was deposited, followed by spin-coating a layer of soap solution [2% Micro-90 diluted in deionized water) before an additional sacrificial 2-μm-thick layer of parylene C (for the subsequent peel-off process) was also deposited. The layers of parylene are then patterned with another layer of positive photoresist (AZ 9260) to shape the PEDOT:PSS electrodes and contact pads. This photoresist is then dry-etched using reactive ion etching to expose electrodes and contact pads. Once etched, a thin film of PEDOT:PSS is spin-coated onto electrodes. The solution is spin-coated three times with soft bakes for 60 s at 120°C. After the final spin-coat, there is a hard bake for 1 hour at 130°C. After baking, the wafer is left over night in DI water to remove excess PSS. The following day, the sacrificial layer of parylene C can be removed, leaving the finished device ready for use. Devices at this stage could either be implanted as control devices or taken through a cell culture protocol to produce a layer of myocytes on fibrin hydrogel before their use in vivo (biohybrid devices).

### Device preparation for cell culture

Custom-made polydimethylsiloxane (PDMS) wells are attached to the MEAs using PDMS as a glue. The devices are plasma-treated at 25 W for 1 min to make the surface hydrophilic for cell culture. The inside of the well is kept wet from this point on with DI water. The devices are entirely sterilized for a minimum of 30 min in 70% ethanol and rinsed with Dulbecco’s phosphate-buffered saline (DPBS).

### Device electrical characterization

Impedance measurements were completed with a potentiostat (Autolab PGSTAT128N) in a three-electrode configuration. An Ag/AgCl electrode was used as the reference electrode, a Pt electrode was the counter electrode, and the working electrode was the recording electrode of the MEA. The characterization was performed in DPBS solution.

### iPSc-derived myocyte cell culture

Human iPSCs were defrosted and expanded in Essential 8 flex medium for approximately 3 to 4 days in six-well plates. This gave approximately 1.5 million cells/ml. They were seeded onto devices with densities of 100,000 cells/cm^2^. Differentiation was initiated 48 hours after cell seeding. The MyoD medium was supplemented with fresh doxycycline (1 μg/ml; Sigma-Aldrich), 1 μM retinoic acid (Sigma-Aldrich), and fibroblast growth factor 2 (40 ng/ml; R&D Systems). The cell culture medium was changed every day from day 0 to day 5. From day 6 onwards, MyoD medium was supplemented only with 1 μM retinoic acid, 3 μM CHIR99021 (Tocris), 10% Knockout serum replacement (KOSR) (Thermo Fisher Scientific), and no doxycycline.

### Fibrin hydrogel preparation

Fibrinogen solution (SOL-FG) was prepared from a fibrinogen stock at a concentration of 37.5 mg/ml in Hepes-buffered saline [HBS; 20 mM Hepes and 150 mM NaCl (pH = 7.4)] by slowly dissolving fibrinogen (F8630, Sigma-Aldrich) for 2 hours at 37°C (solution named SOL-FG). Calcium-thrombin solution (SOL-CaTh) containing thrombin (3 U/ml) and 60 mM calcium ions was prepared by mixing equal volumes of SOL-Th and SOL-Ca, obtaining the solution SOL-CaTh. A solution containing 1,000,000 cells/ml in cell culture media (SOL-Cells) was made. This methodology is used to coat cells that had been grown and differentiated on parylene C–based bioelectronic devices. For the production of fibrin gels, equal volumes of SOL-FG, HBS, and SOL-CaTh, with the cells SOL-Cells, were added on the desired vessels after incubating SOL-FG at 37°C for 2 hours before mixing to allow gelation to occur. Once cells and fibrinogen are mixed, these solutions were used within 15 min, as cells/residual thrombin in cell culture medium will start gelling. Final concentrations are as follows: FG = 3.125, 6.25, 12.5, and 25 mg/ml; Th = 1 U/ml; and CaCl_2_ = 20 mM.

### Animal procedures

All animal work was performed in accordance with the UK Animals (Scientific Procedures) Act 1986. All work was approved by the U.K. Home Office (project license number PFF2068BC) and the Animal Welfare and Ethical Review Body of the University of Cambridge. A total of 190 to 240 g of Hsd:RH-Foxn1rnu athymic nude rats (Envigo, France) were used in this study. Surgical procedures were performed under isoflurane anesthesia, with the animal’s body temperature regulated using a thermal blanket.

For implantation of biohybrid devices, an incision is done over the right forearm, between the shoulder and the elbow of the animal. The triceps muscle is lifted and the forearm nerve bundle (combined ulnar and median nerves) is transected at the height of the elbow. The cell-laden side of a biohybrid implant is then positioned on the cut surface of the nerve and sutured using a 9-0 nylon suture (Ethicon). The implant and nerve are moved toward the midline of the animal and sutured to the latissimus dorsi muscle using two 9-0 nylon sutures, with the forearm nerve facing the muscle and the back of the biohybrid device facing the skin of the forearm. The dermis of the skin above the biohybrid device is scored using a sterile blade, and the incision is finally sutured closed. Control implants consisting of the same flexible MEA design but lacking myocytes are implanted in an identical way. Biohybrid devices are kept sterile throughout the cell culture period and are therefore not sterilized again before implantation. Control devices are sterilized in 70% ethanol for 30 min and rinsed with sterile saline.

For implants later used in terminal recordings (Fig. 3), biohybrid implants including a connecting FFC (flat flex cable) are used. The FFC is folded over once and tucked into a subcutaneous pocket running from the latissimus dorsi to the dorsal cervical region of the animal. Devices are otherwise implanted as above.

For implants later used in awake recordings (Fig. 4), the FFC is run through a subcutaneous tunnel to the head of the animal. A custom-made 3D printed headcap is added as a port of access on the head and fixed using surgical screws and acrylic cement. A screw and a stainless-steel wire are further implanted to connect to the cerebrospinal fluid above the cerebellum and serve as ground during awake recordings. Of the animals implanted for awake electrophysiology, one rat had to be culled past the 3-week time point because of skin damage caused by the device backend connector.

Animals are allowed to recover from the surgical procedure and provided with analgesics (meloxicam and carprofen) for 2 days postimplantation, as well as immediately before surgery. Animals are housed in groups of three or four with ad libitum access to food and water.

### Electrophysiology recordings under anesthesia

Twenty-eight days postimplantation, animals were anesthetized using isoflurane. The FFC of their implanted device is exposed and connected to an electrophysiology acquisition and stimulation system (32-channel RHS headstage and RHS stim/recording controller, Intan Technologies, USA). A pair of platinum wire hooks are implanted into the forearm nerve bundle, first of the contralateral, nonimplanted forearm and then of the operated forearm approximately 4 cm above the point of transection and implantation. The hooks are connected to the same acquisition and stimulation system. A ground shared by the recording MEA and stimulation hook electrodes is implanted into the subcutaneous space of the forearm.

Hooks first implanted in the nonoperated forearm nerve bundle are used to stimulate the nerve (square monopolar pulse, 0.1 ms; 10, 50, 100, or 200 μA), and the amplitude at which a muscle twitch is induced is noted. Hooks are then moved to the implanted forearm nerve bundle. The stimulation paradigm is repeated, with 20 to 30 stimulation pulses delivered for each amplitude in each nerve, and the response is recorded through the biohybrid/control implanted device. Voltage signals are recorded and amplified (X192), band-pass–filtered between 1 and 7.5 kHz, and digitized at a 30-kHz sampling rate. Analysis of the pk-pk amplitude of the response to stimulation in the raw recorded signals is carried out in Spike2 (Cambridge Electronic Design, UK; v9.04b) using a custom script, and plots and statistical tests are carried out in MATLAB (MathWorks, R2021b).

### Awake freely moving rat electrophysiology recordings

Animals are positioned in a 0.3-m by 0.3-m transparent arena. The headcap is opened, and the FFC of the implanted device and the ground are connected to a signal acquisition system. Animals were allowed to roam around the cage while video recordings were taken (GoPro Hero6 Black, GoPro). A green light-emitting diode, driven by a digital out port of the acquisition system, is used to align video and electrical recordings. Videos are manually annotated to indicate stepping events (reaching out and laying weight on the paw of the operated forelimb).

Recorded signals are visualized and quantified in Spike2 software (Cambridge Electronic Design, UK; v9.04b). Biohybrid device signals are produced by referencing pairs of electrodes within the MEA allocated randomly. These referenced signals are then band-pass–filtered (0.5 to 4 kHz; fourth-order Butterworth filter). SNR is calculated as the ratio of the variance during high signal relative to background activity, both identified manually, expressed as decibel. Root mean square (RMS) traces are produced from the referenced and band-pass–filtered signals by calculating the signal RMS at 50-ms intervals and averaging the values using a 0.5-s rolling window.

Normalized signal is calculated by normalizing each RMS trace (single recording session) to range from 0 (background noise) to 1 (highest amplitude signal). The fraction of signal amplitude occurring during step (referred to as correlation) is calculated by comparing the average normalized RMS value during stepping events, relative to the same average value outside of these events. All plotting and statistical comparisons are carried out in MATLAB (MathWorks, R2021b).

### Immunohistochemistry and imaging

Tissue embedding and staining for implanted myocytes (Fig. 2, B and C) occur on a Leica Bond RX autostainer. All steps are performed within a vacuum at 40°C for 1 hour. The steps are as follows: a wash in 70% ethanol and 90% ethanol, four 100% ethanol washes, and three xylene washes, followed by four liquid paraffin wax steps at 63°C. The sections are first baked and dewaxed using a Bond dewax solution (Leica Microsystems, AR9222); then, we move on to the pretreatment protocol where the Bond ER2 solution is their pH 9 antigen retrieval solution (Leica, AR9640) at room temperature. The Bond wash used throughout is AR9590. For the staining protocol, a Bond polymer refine detection kit (Leica, DS9800) was used. The kit includes the peroxidase block, postprimary, and horseradish peroxidase (HRP) polymer secondary antibodies; 3,3′-Diaminobenzidine (DAB); and hematoxylin.

The staining protocol begins with 150 μl of peroxidase block added to the tissue samples and incubated for 5 min at room temperature. The sample is then washed with 150 μl of Bond wash solution three times. Next, 150 μl of the primary antibody, mouse monoclonal to human nucleoli [NM95] (Abcam, ab190710) was used for 60 min at room temperature. The sample is then washed with 150 μl of Bond wash solution three times. One hundred fifty microliters of the postprimary solution is incubated for 8 min at room temperature. Three 150 μl of further Bond washes are performed. Next, 150 μl of HRP polymer secondary antibodies was incubated for 8 min at room temperature. A 2-min incubation with 150 μl of Bond wash solution is performed followed by a wash with 150 μl of DI water and two washes with 150 μl of DAB refine solution. One hundred fifty microliters of hematoxylin is added and incubated for 5 min, followed by washes with 150 μl of DI water, 150 μl of Bond wash solution, and 150 μl of DI water. Samples are then ready to be imaged. Image analysis is performed in ImageJ software (National Institutes of Health). The edge of the tissue facing the device is traced by the user by hand and subsequently unfolded to become a flat image. Color deconvolution is run to separate the implanted cells of interest (brown stain) from the host cells (blue) by difference in histology stain color. The stain intensity values are then imported into MATLAB (MathWorks, R2021b) to produce a mean intensity over distance from the implant using a custom script. Following this, a 400-pixel by 400-pixel box is chosen in the original image in a region of tissue far away from the device, and the average background stain intensity is measured. The intensity profile is divided by this value to produce a normalized intensity for each stain.

Immunofluorescence (Fig. 2, F and G) is carried out by performing antigen retrieval in citrate buffer for 20 min followed by blocking samples in a PBS solution of 5% donkey serum (D9663, Sigma-Aldrich) and 0.1% sodium azide at room temperature of 1 hour. Samples are then stained with anti–b3-tubulin (1/1000 dilution in blocking medium; ab7751, Abcam) and anti–acetylcholinesterase (AChE) (1/100 dilution; ab183591, Abcam). After three washes in blocking medium, the samples were incubated in secondary antibodies [donkey anti-rabbit immunoglobulin G (H + L) highly cross-adsorbed secondary antibody, Alexa Fluor 555, and anti-mouse Alexa Fluor 488; Invitrogen) for 3 hours at room temperature, followed by the Vector TrueVIEW autofluorescence quenching kit (Vector Laboratories) for 3 min, mounted and imaged in an Axioscan slide scanner (Zeiss). Images are then analyzed in ImageJ by performing a thresholding step over the AChE stain images and counting NMJs in an automated fashion using the Analyze Particles tool over an area of approximately 1 mm^2^ in the immediate vicinity to the implanted device. Implanted devices are located through autofluorescence of the parylene C in the 4′,6-diamidino-2-phenylindole channel. All graph plotting and statistical comparisons are carried out in MATLAB.

## Notes

View/request a protocol for this paper from *Bio-protocol*.
