# Supplementary material for: Functional neurological restoration of amputated peripheral nerve using biohybrid regenerative bioelectronics
Source: Sci Adv. 2023 Mar 22;9(12):eadd8162. doi: 10.1126/sciadv.add8162 (PMC10032597; doi:10.1126/sciadv.add8162)
Supplement: Supplementary file 1 — Figs. S1 to S7 [file sciadv.add8162_sm.pdf]

Science Advances
AAAS

Supplementary Materials for

Functional neurological restoration of amputated peripheral nerve using biohybrid regenerative bioelectronics

Amy E. Rochford *et al.*

Corresponding author: Damiano G. Barone, dgb36@cam.ac.uk; George G. Malliaras, gm603@cam.ac.uk

Sci. Adv. **9**, eadd8162 (2023)
DOI: 10.1126/sciadv.add8162

This PDF file includes:

Figs. S1 to S7

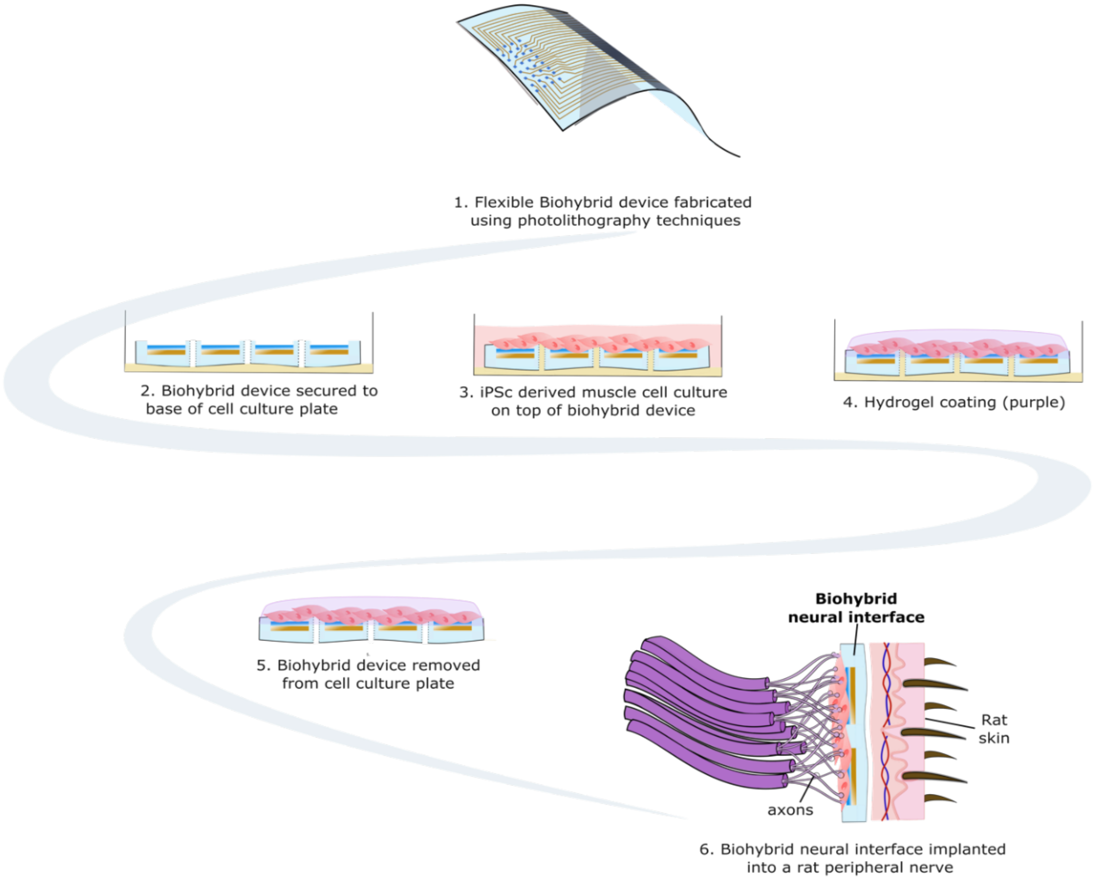

Fig. S1. Biohybrid device *in vitro* culture.

Schematic showing the step by step process involved in culturing human iPSC derived muscle onto biohybrid neural interfaces. Firstly, thin film devices are fabricated using photolithography techniques. These devices are then temporarily adhered to cell culture plates for human iPSC derived muscle culture. Once the muscle are mature a fibrin hydrogel is polymerised on top of the device and cells to ensure they are not damaged during surgical implantation. The device is then removed from the cell culture plate and implanted into the rat peripheral nerve.

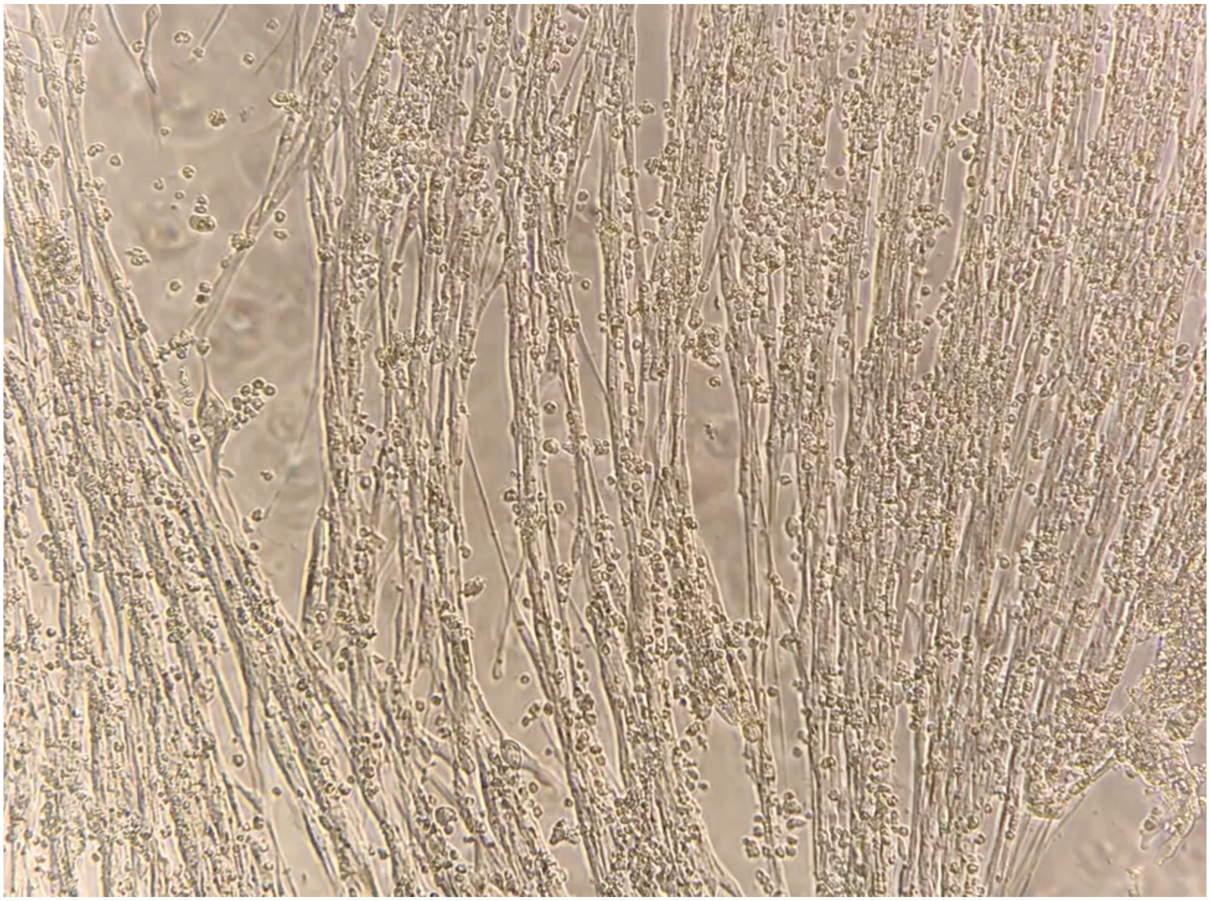

Fig. S2. Differentiated myocytes on a biohybrid device.

Video recording of iPSC derived myotubes contracting at Day 8 of culture after 2mM of acetyl choline was used to stimulate. Recorded using an ECHO Revolve 4 in 1 microscope, 12 MP Colour Camera (Brightfield) at x20 magnification.

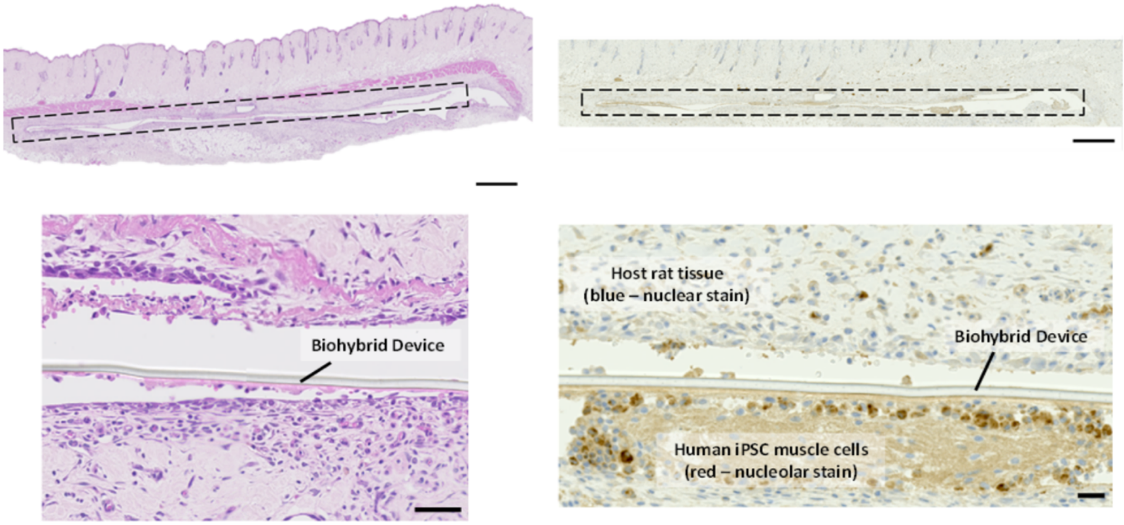

Fig. S3. Human iPSC derived muscle cell survival 7 days post-implantation.
Human iPSC derived muscle cell survival 7 days post-implantation. Left-top, H&E stained ParC sheet coated in human iPSC derived muscle. The dotted line highlights the location of the ParC below the rat skin. Scale bar: 1000µm. Left-bottom, H&E staining of human muscle cells seeded on parylene C sheets after paraffin embedding. The ParC remains intact and a thin layer of cells can be seen on the surface of the device. Right, Paraffin embedded samples with human muscle cells are stained red (using a human nucleoli dye) to confirm survival post-implantation and host rat cells remain in blue.

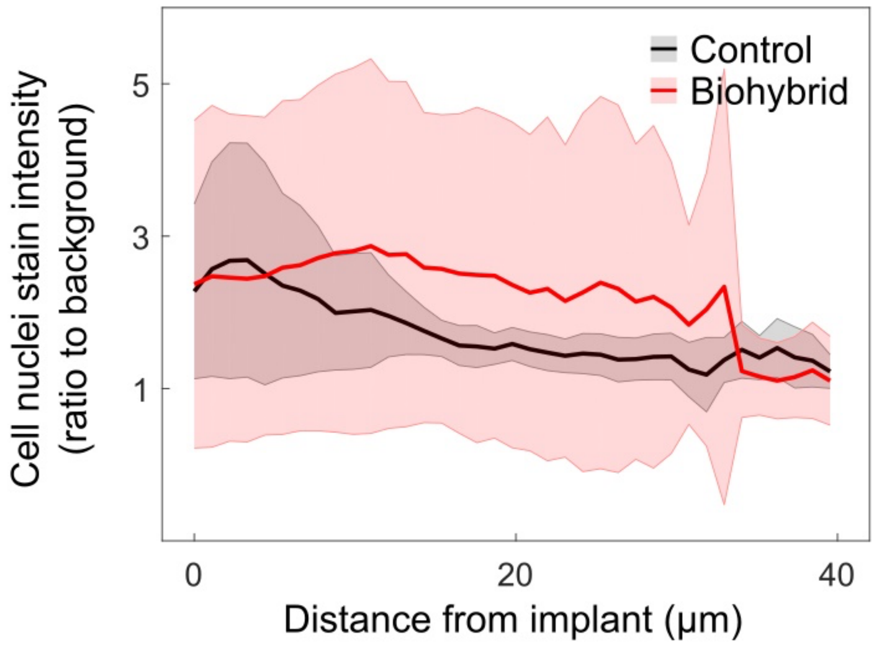

Fig. S4. Quantification of cell density around implanted biohybrid devices.
Cell nuclear stain (blue in Fig. 2B-C) intensity (ratio to background) over distance from 28 day-implanted biohybrid (red) and control (black) implants (mean ± SD). All implants show an increased cellular density close to the implant surface.

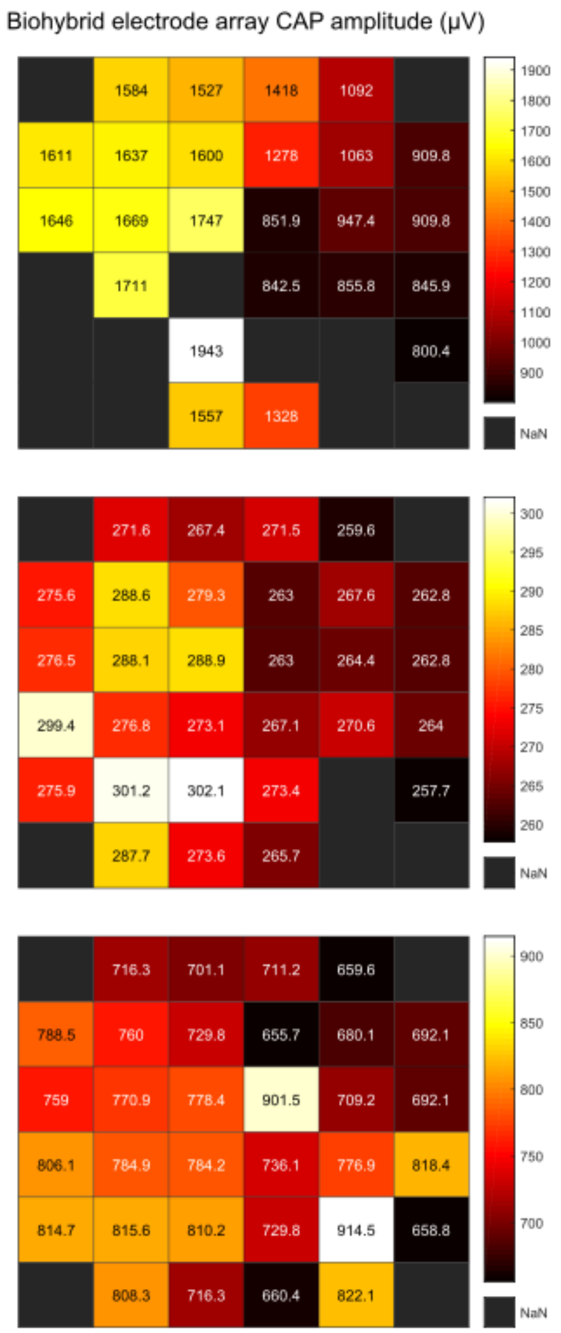

Fig. S5. CAP recording amplitudes on biohybrid MEA.

Average pk-pk CAP amplitude from the MEAs of all three biohybrid devices from Fig. 3. Higher CAP voltages represented by lighter colours in the heatmap, with exact values represented by the numbers. Disconnected (impedance >500 kΩ) are considered disconnected.

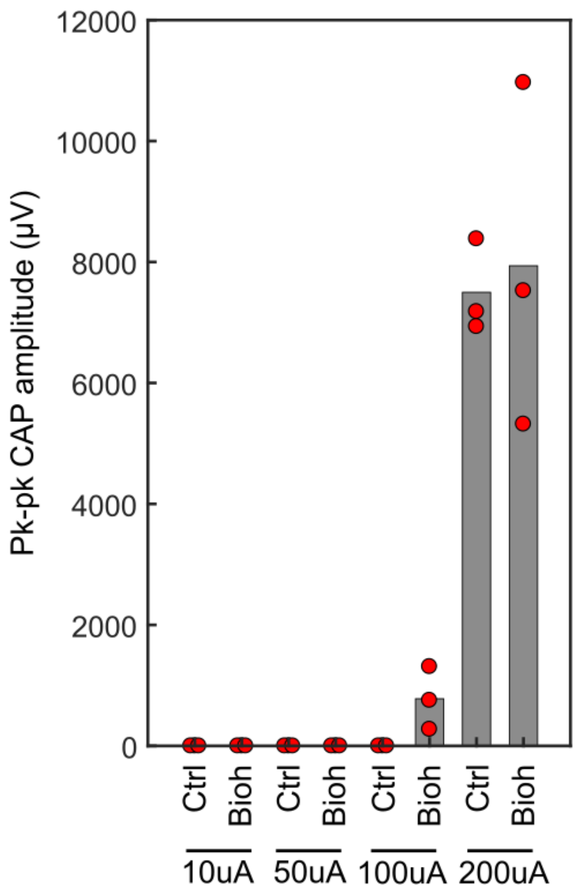

Fig. S6. Quantification of pk-pk CAP amplitude in 28 day-implanted biohybrid and control devices for 0.1 ms pulses of a range of amplitudes.

Quantification of pk-pk CAP amplitude in 28 day-implanted biohybrid and control devices for 0.1 ms pulses of a range of amplitudes. Red circles represent values for each animal (mean across entire MEA), bar indicates mean of whole group.

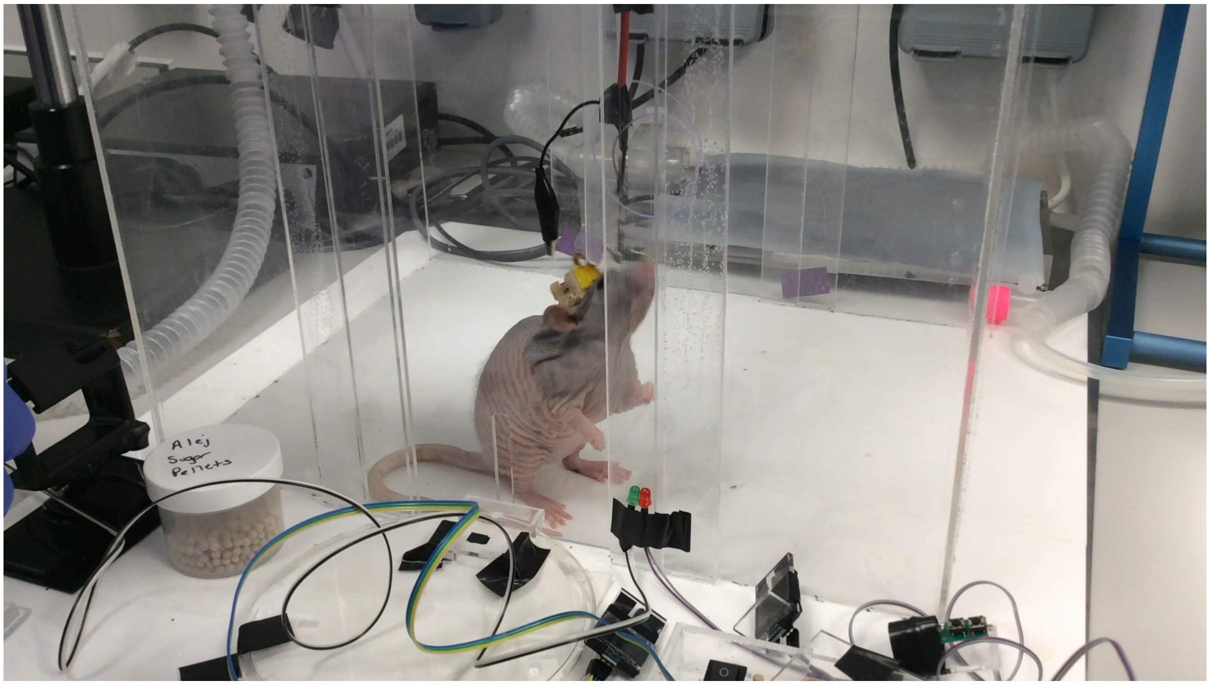

Fig. S7. Awake recording setup.

Sample video of rat during awake recordings from implanted biohybrid device (week 4 post-implantation).
